# Mechanical evaluation of the “Hüfner hand” prosthesis

**DOI:** 10.1177/0309364620952900

**Published:** 2020-09-09

**Authors:** Gerwin Smit

**Affiliations:** Delft University of Technology, Delft, The Netherlands

**Keywords:** Upper limb, prosthesis, cineplasty, Sauerbruch, direct muscle attachment

## Abstract

Current body-powered hands have very low acceptance rates. They also require high activation forces. In the past, a high acceptance rate was reported for the then-available Hüfner hand, a hand which could be controlled by relatively low activation forces.

**Objective:**

The aim of this study was to measure and quantify the mechanical performance of the Hüfner hand.

**Study design:**

Mechanical evaluation.

**Methods:**

Two versions of the Hüfner hand were tested using a mechanical test bench. Forces and displacements were measured under four different glove conditions (no glove, leather, polyvinyl chloride (PVC), silicone). The measured results were compared to data from currently available voluntary-closing hands.

**Results:**

The Hüfner hand required 132–170 Nmm of work and 78–104 N cable force to pinch 15 N. The overall mechanical performance of the Hüfner hands is better than currently available body-powered hands.

**Conclusion:**

The mechanical performance of the Hüfner hand was measured and quantified. Mechanical testing results show that the Hüfner hand has better mechanical performance than current body-powered hands. This may have contributed to its reported high acceptance rates. The design of the Hüfner hand, combined with data presented in this study, can serve as guidelines for the design of a new generation of body-powered hands.

## Background

Recent decades have shown exciting developments in the field of upper limb prosthetics. Multi-articulating hands have become available.^1^ New surgical procedures like targeted muscle reinnervation^2^ and osseointegration have been introduced,^3^ allowing for new control and attachment interfaces.^4^ Despite these impressive developments, body-powered terminal devices can compete well with more complex systems, in terms of speed and functionality.^5^ This was demonstrated at the Cybathlon 2016 competition in Zürich, where the body-powered TRS Grip Prehensor outperformed advanced myoelectric terminal devices.^6^ The voluntary closing (VC) Grip Prehensor is known for its high mechanical efficiency, enabling a high pinch force at a low activation force (i.e. cable force).^7^ The high mechanical performance of the Grip Prehensor stands in sharp contrast to currently available body-powered hands, which have such a low mechanical efficiency that they are of little practical utility. Current body-powered hands require actuation forces that are uncomfortably high and provide low pinch forces in return.^7–9^ Their poor mechanical performance seems to be an important factor in the low acceptance rates of body-powered hands, reported throughout literature, for example, ∼20% by Millstein et al.,^10^ ∼10% by Kejlaa^11^ and 35% by Biddiss et al.^12^

Acceptance rates of body-powered hands have been reported to be higher in the past than in recent years. For example, Lodes et al.^13^ reported an acceptance rate of 70% (*n* = 300) in 1966. This acceptance rate, as well as the research sample, is quite large. Later literature has reported much lower acceptance rates for newer types of body-powered hands.^10–12^ Although multiple factors might have contributed to the high acceptance rates in 1966 (e.g. the limited availability of other options), it would be interesting to measure the mechanical performance (e.g. actuation force) of one type of hand that was used at that time, the Hüfner hand, relative to prosthetic hands that are currently available.

**Figure 1.**
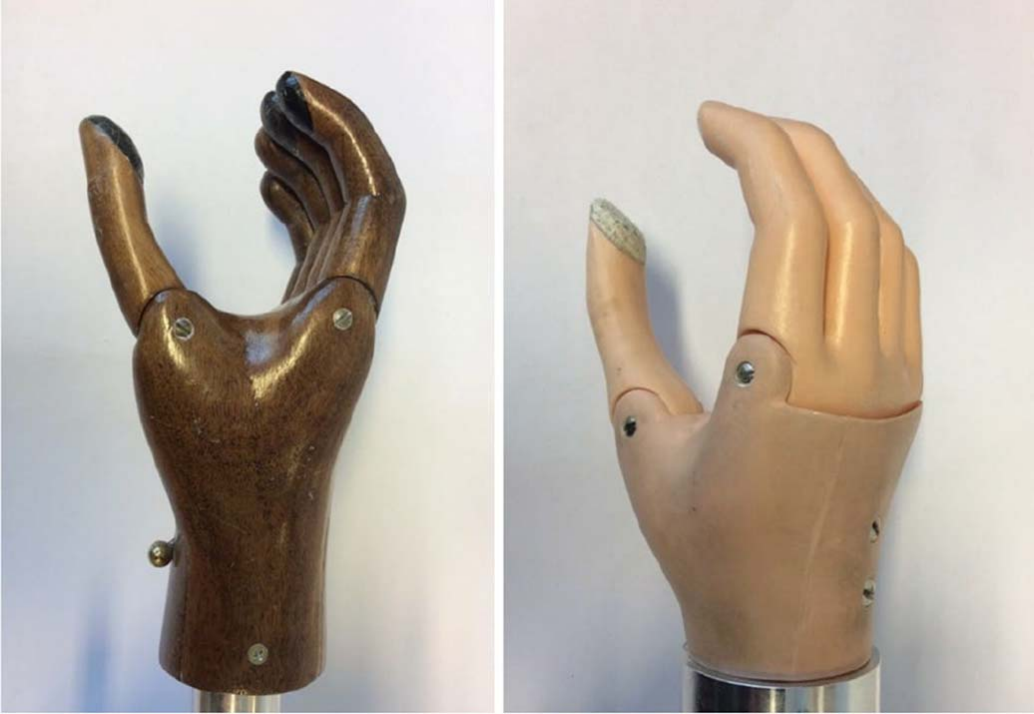
Original wooden Hüfner hand (left) and plastic Hüfner hand (right). The pawl of the locking mechanism can be seen on the palm side.

The Hüfner hand was developed by Jacob Hüfner (1874–1968) in Germany, by the end of the First World War.^14^ It is a VC hand (Figure 1). The hand can be locked in the closed position by means of a ratchet mechanism, patented by Hüfner in 1922.^15^ The lock can be engaged before or during grasping. The locking mechanism is designed in such a way that it can be unlocked with little effort, even when the mechanism is loaded with a very high force. The control pawl of the locking mechanism sticks out from the inside of the palm of the hand. The hand was used for decades as part of the Sauerbruch arm was supplied by Deutsche Ersatzglieder Werkstätte Sauerbruch (DERSA) in Germany.^16^ The Hüfner hand was used as the prototype of the first myoelectric controlled hand, first described by Reinhold Reiter in 1948.^17^ The original Hüfner hand was made of wood. By the end of the 20th century, a plastic model became available (Figure 1) and was produced by VEB Medizinmechanik Suhl/BT Königsee, Germany.^18^

The Hüfner hand was mostly used in combination with control by cineplasty, as were the hands in the study by Lodes et al.^13^ Cineplasty, also known as ‘Direct Muscle Attachment’,^19^ is a surgical procedure in which a mechanical muscle interface is created to control a prosthesis.^14,20–23^ In the procedure, a tunnel is created through a muscle, commonly the biceps. The inside of the tunnel is covered with skin.^24^ After healing and training, the muscle tunnel can be used to control a prosthetic device. A small rod is placed in the tunnel and attached to the actuation cable of the hand. The user can then actuate the hand by contracting the muscle. The muscle interface also provides the user with force and position feedback.^19,25,26^ The maximum forces that can be exerted with a cineplastic muscle tunnel^27,28^ are comparable or lower than the maximum forces that can be exerted by shoulder control.^9^ It is interesting that Lodes et al.^13^ reported high acceptance rates, despite the limited maximum force that can be generated by cineplastic control. That the Hüfner hand is both controllable by cineplasty and is readily accepted by users, suggests that the hand requires relatively low actuation force. However, the mechanical performance of this hand has not been well characterized in recent literature.

The goal of this study was to measure the mechanical performance of the Hüfner hand and compare those results to previously published performance data from other terminal devices available today. We hypothesized that the Hüfner hand would have a lower activation force than other available terminal devices. Results of this study may inform the design of future body-powered hands.

## Methods and materials

Two hands were tested in this study (Figure 1). The first was the original Hüfner hand, made of wood (size 8″). The second hand, made of plastic (size 7¼″), is based on the same design. The hands for this study were new and provided from the stock of OrthoVital (Leipzig, Germany).

**Figure 2.**
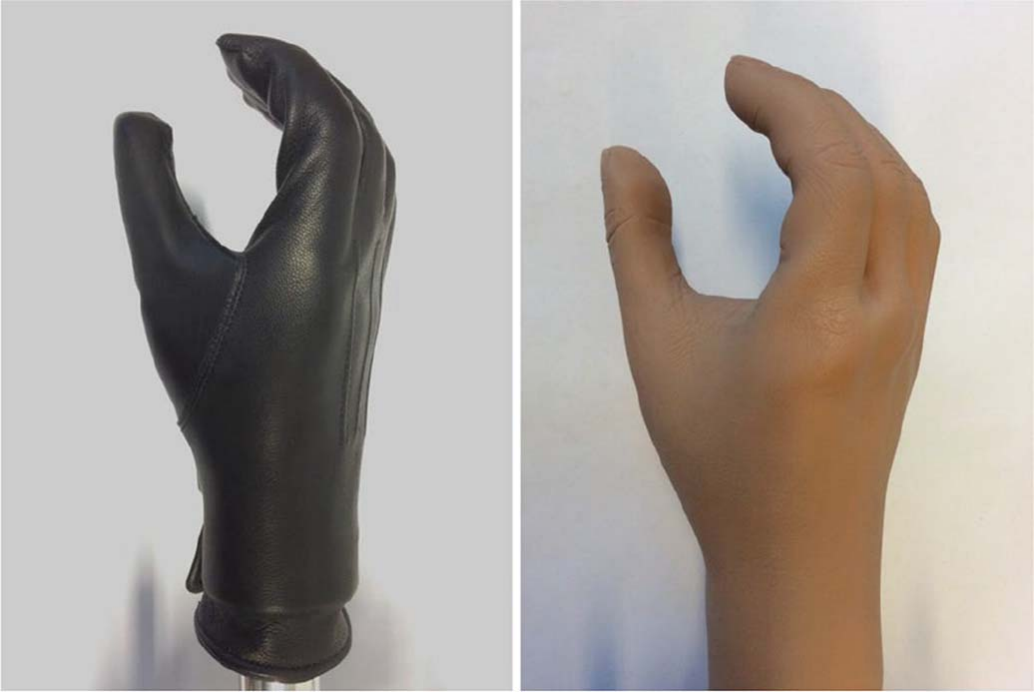
The Hüfner hand was commonly used with a leather glove applied (left). With a contemporary cosmetic glove (right), the Hüfner hand looks similar to a standard myoelectric or body-powered hand.

The hands can be used with different types of gloves. The wooden Hüfner hand was usually used with a leather glove (Figure 2), but could be used without a glove (Figure 1). The hands can also be fitted with a cosmetic polyvinyl chloride (PVC) or silicone glove (Figure 2), giving the hand a similar appearance to a standard myoelectric or body-powered hand. To enable control of the locking pawl, it is necessary to make a hole in the cosmetic glove, on the palmar side.

Past literature has reported that a cosmetic glove^7^ and its material^29^ can have a significant influence on the mechanical behaviour, and hence the required input forces of a prosthetic hand. The hands were therefore tested for four conditions: without a glove, with a leather glove (tailor-made for each hand size), with a silicone glove (SG307 and SG303; RSL Steeper, UK) and with a PVC glove (8S4 = 190 × 76R4 and 8S4 = 210 × 78R4; Otto Bock, Germany).

### Apparatus

The hands were tested using a custom tensile testing bench with a load cell (FLB3G-C3-50kg-6B; Zemic, Etten-Leur, The Netherlands) to measure the forces on the actuation rod of the hand, and a linear variable differential transformer (LCIT 2000; Schaevitz Sensors, Hampton, VA, USA) to measure the displacements of the actuation rod, when opening and closing the hand.^7^ Pinch forces were measured using a custom-built double leave strain gauge load cell (thickness: 10 mm). The signals of the load cell were amplified by a measurement amplifier (CPJ; Scaime, Annemasse, France). All data were recorded using a data acquisition system (NI USB-6008; National Instruments, Austin, TX, USA).

### Procedure

In order to enable a comparison with current VC devices, the mechanical evaluation protocol described by Smit and Plettenburg^7^ was followed. The following parameters were measured: hand mass, glove mass, maximum opening width, maximum cable excursion, work needed for closing the hand, hysteresis of one cycle (closing and reopening), work needed for closing the hand and pinching 15 N, cable force needed to generate a pinch force of 15 N and generated pinch force at a cable force increasing from 0 to 100 N.

Three tests were performed according to the protocol:

1. *Closing test.* The hand was entirely closed and reopened, without pinching and without the pinch force sensor between the fingers.
2. *Pinch test.* The hand was closed until it pinched the pinch force sensor (thickness: 10 mm) with 15 N. Subsequently the hand was reopened.
3. *Pull test.* The hand was closed, with the pinch force sensor (thickness: 10 mm) between the fingers, until the cable pull force reached 100 N.

The closing and pinch tests were repeated four times for the varying glove conditions. The pinch force, activation force and displacements were measured during each test. Work and hysteresis were calculated according to equation (1), in which the cable force (*F*) is integrated along its path (*x*), which is the displacement of the cable (*l*).^7^ The hysteresis, the input work during closing minus the output work during reopening, is a measure of the efficiency. The lower the hysteresis, or dissipated energy, the higher the efficiency. The pull test was not repeated as the variations in test results of repeated tests were expected to be negligible.^7^


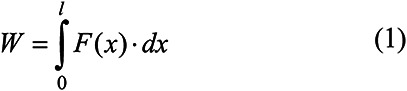


## Results

Table 1 presents the results of the mechanical testing with the Hüfner hands. The values of both hands were in the same range. There were some differences, for example, weight and opening width, due to the different sizes and materials of the hands. The table shows that the addition of a glove decreased the opening width, due to the thickness of the gloves at the fingertips. The gloves increased the required actuation force, work and hysteresis, but the differences between the different gloves were minor.

**Table 1.**
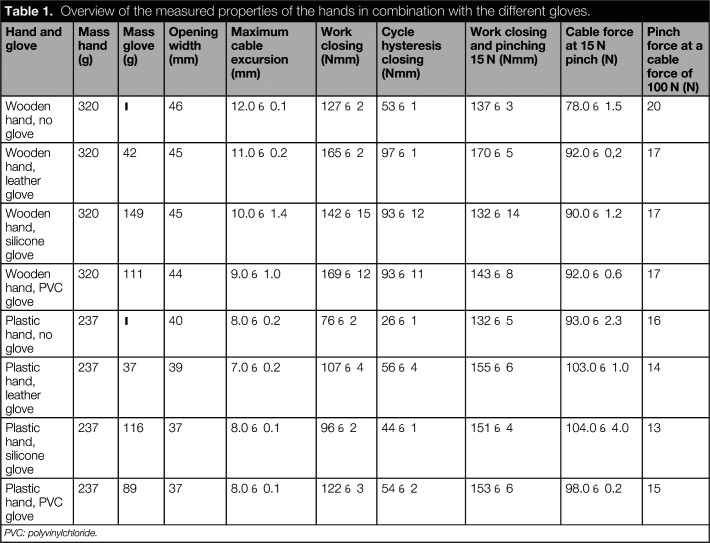
Overview of the measured properties of the hands in combination with the different gloves.

Hand and glove	Mass hand (g)	Mass glove (g)	Opening width (mm)	Maximum cable excursion (mm)	Work closing (Nmm)	Cycle hysteresis closing (Nmm)	Work closing and pinching 15 N (Nmm)	Cable force at 15 N pinch (N)	Pinch force at a cable force of 100 N (N)
Wooden hand, no glove	320	–	46	12.0 ± 0.1	127 ± 2	53 ± 1	137 ± 3	78.0 ± 1.5	20
Wooden hand, leather glove	320	42	45	11.0 ± 0.2	165 ± 2	97 ± 1	170 ± 5	92.0 ± 0,2	17
Wooden hand, silicone glove	320	149	45	10.0 ± 1.4	142 ± 15	93 ± 12	132 ± 14	90.0 ± 1.2	17
Wooden hand, PVC glove	320	111	44	9.0 ± 1.0	169 ± 12	93 ± 11	143 ± 8	92.0 ± 0.6	17
Plastic hand, no glove	237	–	40	8.0 ± 0.2	76 ± 2	26 ± 1	132 ± 5	93.0 ± 2.3	16
Plastic hand, leather glove	237	37	39	7.0 ± 0.2	107 ± 4	56 ± 4	155 ± 6	103.0 ± 1.0	14
Plastic hand, silicone glove	237	116	37	8.0 ± 0.1	96 ± 2	44 ± 1	151 ± 4	104.0 ± 4.0	13
Plastic hand, PVC glove	237	89	37	8.0 ± 0.1	122 ± 3	54 ± 2	153 ± 6	98.0 ± 0.2	15

PVC: polyvinylchloride.

### Closing test and pinch test

**Figure 3.**
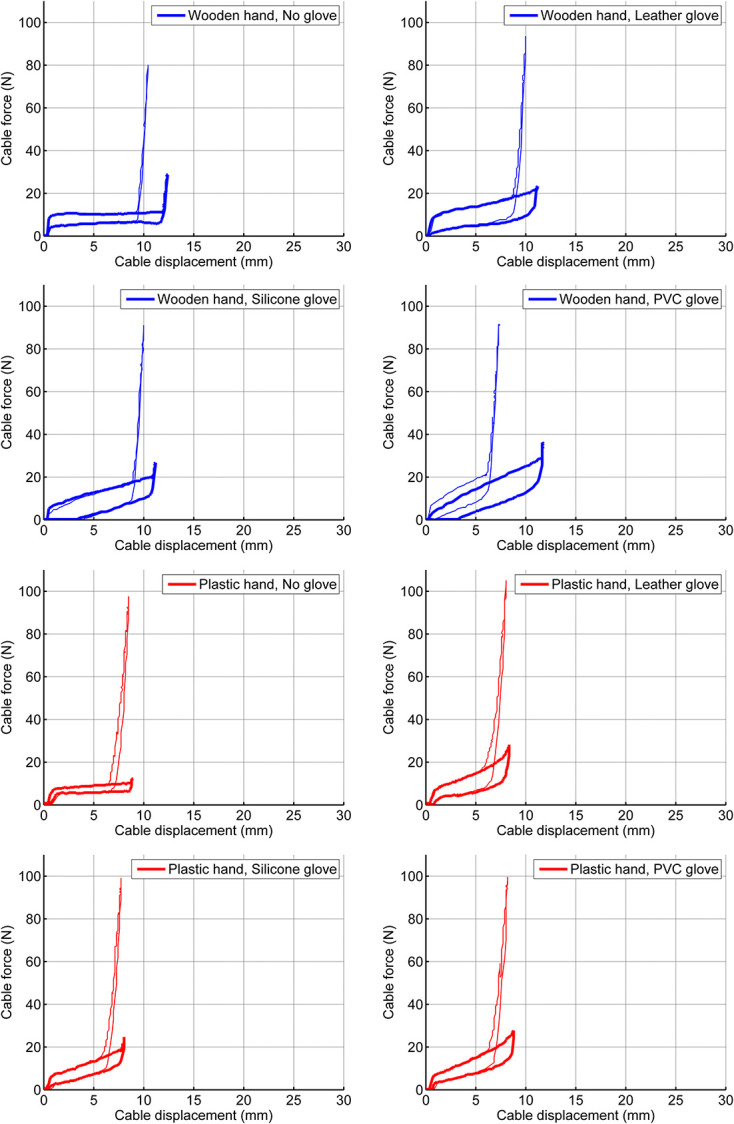
Closing and pinch test results with the Hüfner hand. The thick lines represent the results of the closing test, in which the hand was fully closed. The thin lines represent the results of the pinch test, in which a pinch force sensor (*t* = 10 mm) was pinched between the fingers, until a pinch force of 15 N was reached. The diagrams of the pinch test show a smaller displacement, due to the thickness of the sensor between the fingers, and they show a higher cable force, due to the required pinch force. Each test was repeated four times. All cycles look similar. For clarity, only one of the four cycles is presented.

**Figure 4.**
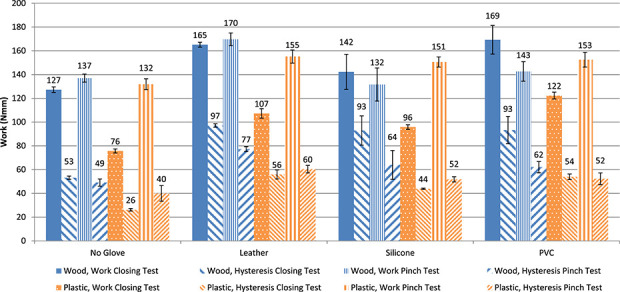
Work and hysteresis associated with the Hüfner hand during the closing and pinch tests. Each test was repeated four times. The graphs show the mean values, for the closing test and for the 15-N pinch test. Error bars indicate the standard deviation for each series of tests.

**Figure 5.**
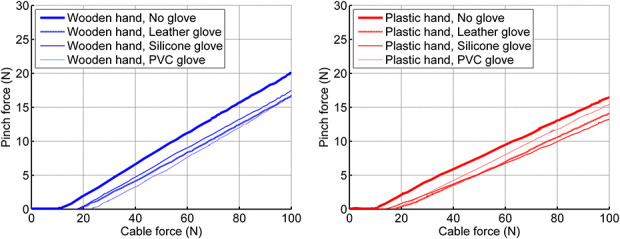
Results of the 0–100 N pull test with the Hüfner hand show the pinch force as function of the cable force. A cable force between 0 and 25 N is required to close the hand, to touch the pinch force sensor and to start building up a pinch force. The linear relation between cable force (or actuation force) and pinch force represents the transmission ratio.

The measured displacement force diagrams of the closing and pinch test are presented in Figure 3. The thick lines represent the results of the closing test, without pinching. The thin lines represent the results of the pinch test, in which a pinch force sensor (*t* = 10 mm) was pinched between the fingers, until a pinch force of 15 N was reached. The diagrams of the pinch test show a smaller displacement than the ones of the closing test. The hand closes less in the pinch test, due to the thickness of the sensor between the fingers. Hands with a glove require a higher cable force to pinch 15 N in the pinch test, due to the resistance of the glove. The area enclosed by the diagrams represent the hysteresis of the hand. Hands with gloves have a larger enclosed area, which means a larger amount of dissipated energy, thus a lower energy efficiency of the system. Figure 4 shows the calculated work and hysteresis for both tests. The force displacement curves (Figure 3) show that the stiffer PVC glove requires slightly more force than the more flexible silicone glove. Nevertheless, the characteristics are quite similar for the different glove materials (see also Figures 4 and 5).

### Pull test

Figure 5 shows the pinch force as a function of the activation force, for both hands, with the various glove conditions. The hands require a force of 10–25 N to close the hand and then begin to build up a pinch force. The slope of the curve represents the transmission ratio.

## Discussion

### Activation force and pinch force

A terminal device should be able to generate sufficient pinch force (>15 N) to accomplish a broad range of daily tasks at a comfortable activation force level.^7^ Preferably, a device should allow for higher pinch forces (>30 N).^30^ Repeating activation forces should preferably be at a fatigue-free level, which is estimated to be below 66 N for males and below 38 N for females.^9^ Figure 3 and Table I show that the measured activation forces are above the proposed fatigue limits (90–104 N). Figure 3 also shows something remarkable, the required cable displacements for closing the hands (∼10 mm) are three to five times smaller than those of current terminal devices (37–49 mm).^7^ This means that there is potential cable displacement that can be used, either by changing the hand mechanism or by adding a simple lever transmission to the system. Such a lever could be placed inside the shaft of the prosthesis’ forearm. The lever would amplify the cable displacement and would reduce the required actuation force by the same amplification factor. A lever with a transmission ratio of 3 would increase the cable displacement three times (∼10 mm × 3 = ∼30 mm), creating a cable displacement closer to that of current hands. At the same time, such a lever would reduce the cable force three times (104 N/3 = 35 N), enabling both men and women to generate a pinch force of over 15 N repeatedly without fatigue.

### Comparison to other hands

**Figure 6.**
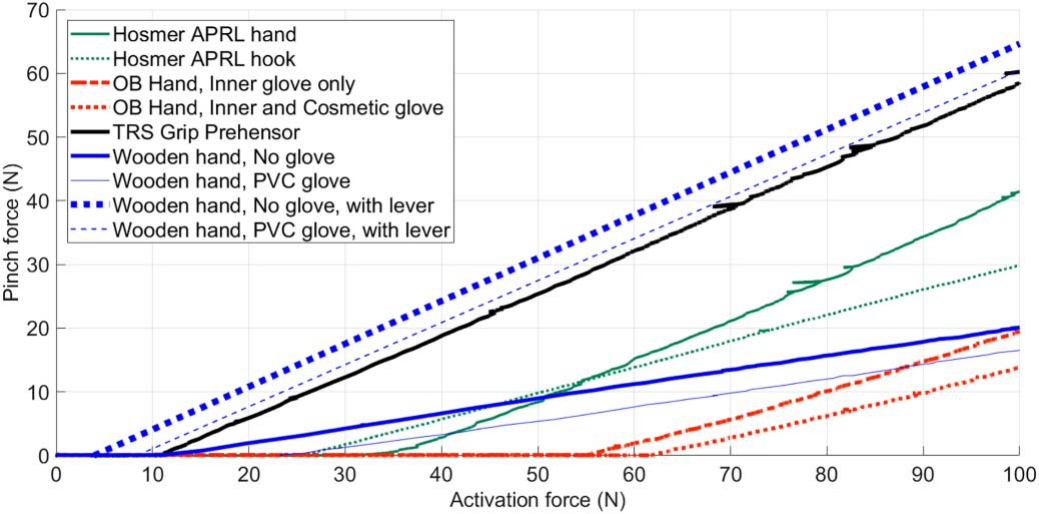
The characteristics of activation force, or cable force, and pinch force of the wooden Hüfner hand, compared to currently available body-powered terminal devices (adapted from Smit and Plettenburg^7^). The dashed lines at the left show the estimated effect of using a cable displacement amplification lever, with a transmission ratio of 3. The slope of the curve is determined by the transmission ratio. The intersection of the *x*-axis indicates how much force in necessary to close the hand and start pinching. This is predominantly determined by the strength of the opening spring.

The measured Hüfner hands (320/237 g) have a lower mass than other VC hands, like the APRL hand (347 g), the Otto Bock VC hand (350 g) and the Hosmer VC Soft hand (366 g). The opening span of the Hüfner hands (46/40 mm) is similar to that of the APRL (44 mm, with standard thumb setting), but smaller than that of the Otto Bock VC hand (69 mm) and the Hosmer VC Soft hand (71 mm).^7^ This means that the Hüfner hand would need a re-design in order to pick up larger objects.^24^ The Hüfner hand has a lower transmission ratio (input force/output force = ∼ 0.23) than the APRL hand (0.65), the Otto Bock hand (0.36) and the TRS Grip Prehensor (0.66) (see Figure 6). The Hüfner hand requires a lower activation force than the Otto Bock VC hand. Apparently, the opening spring of the Hüfner hand exerts a lower force than the Otto Bock hand, when the hand is closed. The activation force would be improved even further when a transmission lever would be applied.

Figure 6 shows the results of the current study, combined with those from the study by Smit and Plettenburg on contemporary VC devices.^7^ The blue dotted lines in Figure 6 shows the estimated characteristics of the activation force and the pinch force of the wooden Hüfner hand combined with a transmission lever at a ratio of 3. In this configuration, both the ungloved and gloved Hüfner hands can produce a higher pinch force than contemporary devices. These force values were calculated without considering friction. In practice, a transmission lever will add some friction and the required activation force will be somewhat higher. The characteristics of the Hüfner hand and a transmission lever with friction are expected to yield characteristics similar to that of the TRS Grip Prehensor, requiring an activation force which is 25 N lower than that of the APRL hand. Increasing the transmission ratio to 4 could further reduce the required activation force of the Hüfner hand and bring it below the force level of the TRS Grip Prehensor. For a transmission ratio of 4, the required cable displacement of the Hüfner hand (∼10 mm × 4 = ∼40 mm) would still be smaller than that required by the TRS Grip Prehensor (49 mm). It should be noted, however, that the opening span of the Hüfner hands (46/40 mm) is smaller than that of the TRS Grip Prehensor (72 mm).

Compared to the hands tested by Smit and Plettenburg,^7^ the work and hysteresis required to operate the Hüfner hand (Figure 4) are remarkably low. Closing and pinching without a glove with the Hüfner hand requires one-twelfth of the energy (137 and 132 Nmm) required by the Otto Bock hand (1694 Nmm).^7^ This might be attributed to the absence of an inner glove in the Hüfner hand, and to its smaller opening span (46 mm) than the Otto Bock hand (69 mm). Comparison with the APRL hand is even more remarkable. The APRL hand has the best mechanical performance of currently available VC hands.^7^ It has no inner glove, and its opening span is the same as the Hüfner hand, yet the APRL hand requires six times more energy (831 Nmm) for closing and pinching. This is noteworthy since, like the Hüfner hand, the APRL hand was designed to be compatible with cineplastic operation but it is newer. It is not clear what causes the differences between these hands, although Figure 6 shows that the Hüfner hand uses a lower spring tension to open the hand, compared to the other devices. It is noteworthy that newer devices like the Otto Bock hand, the APRL hand and even the APRL hook require more input energy than the Hüfner hand. The superior mechanical performance of the Hüfner hand might have been an important factor in the high acceptance rate of this hand in a prior study.^13^

### Effect of the gloves

The effect of the different outer gloves is limited (Figures 3–5). This is unexpected, as PVC and silicone gloves typically have a very different stiffness.^29^ A likely explanation for the small differences is that the gloves do not stretch so much when the fingers flex. When the fingers flex, the dorsal surface of the moving joints increases (at the knuckles), while the palmar area of the moving joints, and that of the purlicue (skin between thumb and index finger) decreases. To accommodate these changes, the glove material can stretch and unstretch, or unwrinkle and wrinkle (or both). Stiffer materials require more force for stretching than flexible materials. In general, stretching requires more force than wrinkling. In the gloves applied to the Hüfner hands, there seems to be more wrinkling than stretching. Therefore, the undesired counteracting force of the (outer) gloves is limited and the effect of the material stiffness is minor. The Hüfner hand has no inner glove. The study by Smit and Plettenburg^7^ showed that the inner glove of the Otto Bock hand has undesired mechanical properties and hampers smooth and easy operation of the mechanism, as it adds an extra layer of deforming material. The measurement of the Hüfner hand confirms this observation. The absence of an inner glove in the Hüfner hands likely contributes to a higher mechanical efficiency, when compared to the Otto Bock hand.

### Study limitations

As the Hüfner hand is no longer being produced, there were only a few unused hands available for testing. The age of the tested hand specimens may influence performance. Also, only one specimen was tested for each type of hand (i.e. wooden and plastic) and only one specimen was tested per hand for each type of glove (i.e. leather, silicone and PVC). There might be variations in the mechanical properties between hands, due to variations in the production. For gloves, it is known that there can be measurable variations in mechanical properties between gloves.^31^ Changing the transmission ratio by adding an extra lever is expected to lower the actuation force. This is expected to be preferred by the user, but was not tested in this study.

### User acceptance

The reported acceptance rates of body-powered hands are very low, for example, ∼20% by Millstein et al.,^10^ ∼10% by Kejlaa,^11^ and 35% by Biddiss et al.^12^ The same studies reported much higher acceptance rates for body-powered hooks, for example, ∼68% by Millstein et al.^10^ and 49% by Biddiss et al.^12^ In Kejlaa’s study, the users preferred the hook over the ‘troublesome’ body-powered hand. They mention that the hand required more power to operate than the hook. In the study by Millstein et al., users reported that the body-powered hand was hard to control and had a weak grip. In contrast to these findings, Lodes et al.^13^ reported an acceptance rate of 70% for body-powered hands in 1966. Hooks played only a minor role in their study. The results of our study, when combined with the acceptance rates in prior studies, suggest that mechanical performance may be a factor affecting acceptance rate of body-powered hands. The Hüfner hand used in the study by Lodes et al. required a low force, whereas the newer hands required much higher forces, as reported in later studies.^7,8^ Beside activation force, there may be many other factors that influence the selection of a terminal device, for example, reimbursement, availability of other options, and preference of the prosthetist. The mode of actuation might also influence acceptance rate. The Hüfner hand was often controlled by cineplasty, which provides the user with good function in a wider range motion of the prosthetic arm than when controlling with a shoulder strap.^32^ The Hüfner hand had its flaws. Its opening span was relatively small and the locking pawl was located in the palm of the hand. This may not be a concern when using a leather glove with an available opening, but a cosmetic glove needs to be punctured, which is undesirable. It is interesting that a prosthetic hand that was available in the past, but is no longer available today, exhibits superior mechanical performance when compared to contemporary, commercially available body-powered hands. Given the potential for body-powered devices to outperform even contemporary myoelectric devices in many real-world activities,^6^ there may be merit to looking at the Hüfner hand as an inspiration for new prosthetic hand designs.

## Conclusion

In this study, the mechanical performance of the Hüfner hand was measured and quantified. Two models, the original wooden model and a later plastic model, were tested with and without gloves. Effects of different glove materials on mechanical performance were minor. The Hüfner hand has a better mechanical performance than current body-powered hands. This may be a factor that contributed to its reported high acceptance rates. The design of the Hüfner hand, combined with data presented in this study, can serve as guidelines for the design of a new generation body-powered hands that are more readily accepted and used by people with upper limb absence.
